# Crowned Dens Syndrome Mimicking Atlantoaxial Infection in a Patient With Systemic Sclerosis: A Case Report

**DOI:** 10.1155/crrh/3673141

**Published:** 2026-02-22

**Authors:** Tarun Selvarajan, Lavanya Kannekanti, Shravya Balmuri, Samina Hayat

**Affiliations:** ^1^ Department of Internal Medicine, Louisiana State University, Shreveport, Louisiana, USA, lsu.edu; ^2^ Department of Rheumatology, Center of Excellence for Arthritis and Rheumatology, LSU, Shreveport, Louisiana, USA

## Abstract

**Objective:**

Crowned dens syndrome (CDS), caused by calcium pyrophosphate or hydroxyapatite deposition around the odontoid process, is an under‐recognized cause of acute severe neck pain and headache. It can closely mimic septic arthritis, osteomyelitis, giant cell arteritis (GCA), polymyalgia rheumatica (PMR), or cervical spondylitis. We report a case of CDS in a patient with diffuse systemic sclerosis initially managed for presumed infection.

**Methods:**

A 55‐year‐old man with diffuse cutaneous systemic sclerosis on mycophenolate mofetil and methotrexate presented with severe throbbing headache, facial tenderness, and diffuse pain. CT/CTA of the head and neck were negative for vascular events; MRI revealed inflammatory changes at the atlantoaxial and atlanto‐occipital joints concerning for septic arthritis/osteomyelitis with abscess formation. Broad‐spectrum antibiotics were initiated.

**Results:**

The patient’s symptoms persisted despite antibiotics, prompting repeat imaging. CT demonstrated calcifications surrounding the odontoid process, raising suspicion for CDS. Colchicine and low‐dose prednisone were introduced while antibiotics were continued, given ongoing concern for occult infection in an immunosuppressed host. Within 1 week, the patient experienced near‐complete resolution of pain. At follow‐up, he remained symptom‐free, allowing reintroduction of methotrexate while mycophenolate was held.

**Conclusion:**

CDS should be considered in the differential diagnosis of severe headache and neck pain in rheumatology patients, particularly when imaging shows craniocervical inflammation and calcifications. CT of the odontoid is diagnostic, but MRI findings may mimic infection. Awareness of CDS is critical to avoid misdiagnosis, unnecessary procedures, or prolonged inappropriate therapy. Prompt recognition and anti‐inflammatory treatment can be rapidly effective and permit safe reintroduction of disease‐modifying therapy.

**Significance and Innovation**
 Crowned dens syndrome (CDS) is an under‐recognized cause of acute cervico‐occipital pain and is frequently misdiagnosed as atlantoaxial infection, often leading to unnecessary antibiotics and invasive procedures. Although CDS has only rarely been described in patients with systemic sclerosis, this case highlights the diagnostic challenge of distinguishing it from atlantoaxial infection in an immunosuppressed patient with connective tissue disease reinforcing the need to consider CDS when such patients present with acute neck pain and normal inflammatory markers.
 We emphasize the pivotal role of noncontrast CT of the craniocervical junction in establishing the diagnosis, as MRI findings alone may mimic infection and easily mislead clinicians. The characteristic of periodontoid calcification on CT is ultimately what differentiates CDS from infectious or inflammatory mimics.
 Finally, our case provides a practical, stepwise diagnostic and therapeutic framework for managing CDS in immunocompromised hosts, balancing the imperative to rule out infection while initiating targeted anti‐inflammatory therapy to achieve rapid symptom improvement.

## 1. Introduction

Acute neck pain and headache in patients with systemic autoimmune disease present a diagnostic challenge. Differential diagnoses often include vasculitides such as giant cell arteritis, central nervous system involvement of connective tissue disease, or infectious etiologies such as atlantoaxial septic arthritis and osteomyelitis [1, 2]. Among these, CDS remains under‐recognized [1]. CDS results from calcium pyrophosphate or hydroxyapatite crystal deposition around the odontoid process and can mimic infection, polymyalgia rheumatica, meningitis, or cervical spondylitis [1, 3, 4]. Awareness of CDS is essential, as its imaging features and inflammatory profile often lead to misdiagnosis and unnecessary antimicrobial therapy. We report a case of CDS in a patient with diffuse systemic sclerosis, highlighting the diagnostic pitfalls, the complementary role of CT and MRI, and the clinical approach in an immunosuppressed host.

## 2. Methods

Ethics approval was not required for this case report, as no identifiable patient information was included. Patient confidentiality and privacy were strictly maintained in accordance with institutional and journal guidelines.

## 3. Case Report

A 55‐year‐old man with hypertension and diffuse cutaneous systemic sclerosis on mycophenolate mofetil and methotrexate presented with four days of severe bilateral occipital headache and neck pain. The pain was throbbing, radiating to the shoulders, and associated with facial tenderness and malaise. He denied trauma, visual symptoms, or neurological deficits. He was afebrile on presentation, with stable vital signs. He had no prior history of acute crystal arthritis, chondrocalcinosis, or radiographic evidence of calcium pyrophosphate deposition in peripheral joints. Examination revealed marked tenderness over the upper cervical spine and restricted neck motion, but no focal neurological deficits or meningeal signs.

**FIGURE 1:**
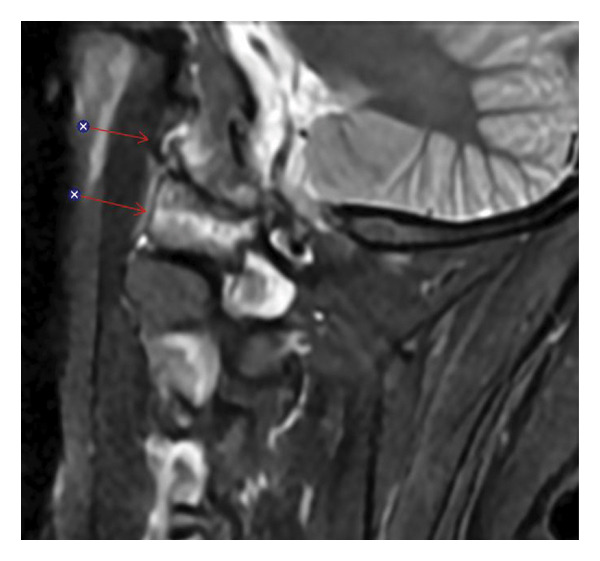
Sagittal STIR MRI of the upper cervical spine showing bone marrow edema around the left atlanto‐occipital joint with associated hyperintense signal in the adjacent soft tissues (arrows).

**FIGURE 2:**
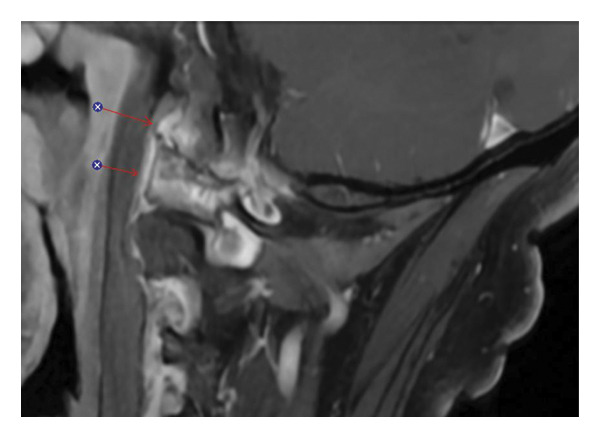
Sagittal fat‐suppressed postcontrast T1‐weighted MRI demonstrating enhancement of the periodontoid soft tissues and left atlanto‐occipital region corresponding to the areas of edema seen on the STIR sequence in Figure 1 (arrows).

Laboratory studies, including C‐reactive protein and erythrocyte sedimentation rate, were unremarkable. Given his presentation, an acute vascular or infectious etiology was initially suspected. CT angiography of the head and neck revealed no vascular abnormalities. Brain MRI was negative for ischemia but showed abnormal enhancement at the C1‐C2 atlantoaxial and atlanto‐occipital joints, with adjacent soft tissue thickening suggestive of early abscess formation (Figures 1 and 2). These findings raised concern for septic arthritis or osteomyelitis.

**FIGURE 3:**
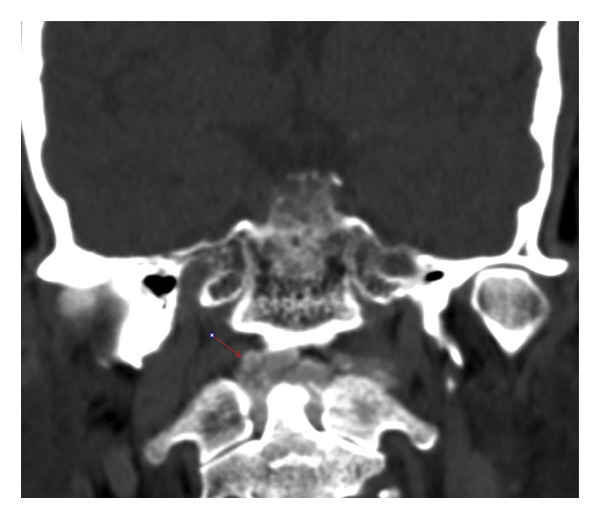
Coronal noncontrast CT of the craniocervical junction demonstrating curvilinear calcium mineralization surrounding the odontoid process, consistent with periodontoid calcification at the C1‐C2 level (arrow).

Empirical broad‐spectrum intravenous antibiotics (vancomycin and cefepime) were initiated. Neurosurgical evaluation was advised against surgical drainage because of high procedural risk. After 48 h, the patient’s pain persisted without improvement. Repeat MRI demonstrated progression of marrow edema and enhancement. Because of the lack of clinical response, a noncontrast CT of the cervical spine was obtained (Figure 3). This revealed a dense ring of calcification surrounding the odontoid process, increasing suspicion for CDS [3].

Rheumatology was consulted, and mycophenolate was discontinued, while methotrexate was continued. Low‐dose prednisone (10 mg daily) and colchicine (0.6 mg twice daily) were started for suspected crystal‐mediated inflammation. Given his immunosuppression and initial MRI findings suggestive of an abscess, the antibiotic regimen was maintained temporarily to mitigate the risk of an untreated infection.

Within 2 days of initiating colchicine and prednisone, the patient reported substantial pain relief. By 1 week, headache and neck stiffness had nearly resolved. Inflammatory markers remained unremarkable, and antibiotics were discontinued. He was discharged on colchicine and a tapering prednisone course. At follow‐up, the patient remained symptom‐free. Serial C‐reactive protein measurements remained within the normal range throughout hospitalization and at outpatient follow‐up, paralleling his clinical response. Mycophenolate was gradually reintroduced, and he continued methotrexate without recurrence of symptoms.

## 4. Discussion

CDS is an uncommon but important cause of acute cervico‐occipital pain. CDS predominantly affects elderly individuals but may occur in immunosuppressed patients with connective tissue disease. Its pathogenesis involves deposition of calcium pyrophosphate or hydroxyapatite crystals in the transverse and alar ligaments [1, 3].

While crowned dens calcification is most commonly associated with calcium pyrophosphate deposition disease, it is not pathognomonic for CPPD and can also occur with other patterns of crystal or dystrophic calcification [3, 5]. In particular, systemic sclerosis is frequently complicated by soft‐tissue and periarticular calcinosis, most often due to hydroxyapatite deposition [6, 7], and several case reports and small series have described cervical spine and ligamentous calcification in this population [8–11]. These observations suggest that crowned dens calcification in patients with systemic sclerosis may represent a broader spectrum of crystal deposition and dystrophic calcification rather than isolated CPPD, and this possibility should be considered when interpreting craniocervical imaging in connective tissue disease.

Clinically, CDS presents with acute neck pain, limited cervical motion, and elevated inflammatory markers, often accompanied by fever. Because these findings overlap with meningitis, cervical discitis, epidural abscess, septic arthritis, osteomyelitis, metastatic cervical spine disease, polymyalgia rheumatica, and giant cell arteritis, misdiagnosis is frequent [2–4]. MRI findings such as marrow edema, enhancement, and joint effusions are nonspecific and often interpreted as infection. In contrast, noncontrast CT of C1‐C2 is diagnostic [3, 12], revealing curvilinear calcifications posterior and lateral to the odontoid. The differential diagnosis of periodontoid calcification therefore includes CPPD‐related CDS, hydroxyapatite deposition disease, and connective‐tissue‐disease–associated dystrophic calcification, as well as less common metabolic and degenerative causes.

In contrast, this patient never developed fever and had normal C‐reactive protein values at presentation and follow‐up, despite marked neck pain and striking inflammatory changes on MRI. This discordance between systemic inflammatory markers and imaging findings can obscure the diagnosis and may falsely reassure clinicians when evaluating for infection in immunosuppressed hosts, emphasizing the need to integrate serial laboratory data with clinical course and repeat imaging. In this case, the patient’s immunosuppressed status and MRI findings of inflammatory enhancement prompted empiric antibiotic therapy; however, the lack of clinical response led to repeat CT which was crucial in identifying periodontoid calcification consistent with CDS. Temporary continuation of antibiotics after the diagnosis was reasonable because the MRI suggested a possible abscess and the risk of missing an occult infection was significant.

Treatment of CDS consists of anti‐inflammatory therapy with NSAIDs, colchicine, or corticosteroids [3, 12]. Clinical improvement typically occurs within several days. Our patient responded promptly to colchicine and low‐dose prednisone, confirming the diagnosis. The rapid resolution of pain even with normal inflammatory markers throughout supported a crystal‐mediated process rather than infection. Once symptoms resolved, his immunosuppressive regimen was safely resumed.

This case emphasizes three critical points. First, CDS should be considered in any patient with acute neck pain, especially if infection or vasculitis has been excluded. Second, CT of the craniocervical junction is the imaging modality of choice, as MRI alone may be misleading. Third, in immunosuppressed individuals, a combined approach with temporary antibiotics and anti‐inflammatory therapy may be necessary until infection is confidently ruled out. Awareness of CDS can prevent unnecessary invasive procedures and prolonged antibiotic courses.

## Funding

The authors received no specific funding for this work.

## Conflicts of Interest

The authors declare no conflicts of interest.

## Data Availability

The data that support the findings of this study are available from the corresponding author upon reasonable request.
